# Reasons for Refusing Home-based Palliative Care: A Qualitative Study of Patients and Caregivers

**DOI:** 10.1093/geroni/igaa057.3326

**Published:** 2020-12-16

**Authors:** Valeria Cardenas, YuJun Zhu, Anna Rahman, Susan Enguidanos

**Affiliations:** University of Southern California, Los Angeles, California, United States

## Abstract

Despite some insurance plans now paying for home-based palliative care, recent reports have suggested that coverage for palliative care may be insufficient to expanding patient access to home-based palliative care. Research has yet to explore palliative care barriers from the perspective of palliative care-eligible patients and their caregivers. To identify patients and caregivers’ perceived barriers to home-based palliative care and their recommendations for overcoming these barriers, we conducted a qualitative study using semi-structured individual interviews. Participants (patients, proxies, and their caregivers) who were eligible for a randomized controlled trial of home-based palliative care were interviewed via telephone. Our interview protocol elicited participants’ perspectives on home-based palliative care services; positive and negative aspects of the program explanation; and suggestions for improving messaging around home-based palliative care. Researchers used grounded theory to identify the themes within the transcripts. Two researchers independently coded the transcripts and then met to compare coding and reconciled discrepancies until 100% consensus was reached. Identified themes related to home-based palliative care referral barriers included reluctance to have home visits, timing, lack of palliative care knowledge, misconceptions of palliative care, and patients’ self-perceived health condition (not sick enough for palliative care). Themes related to recommendations for overcoming these barriers included preferring a palliative care referral from healthcare providers or from insurance company and clearer presentation of palliative care service. Findings reinforce the need for additional palliative care education among patients with serious illness and the importance of delivering the information from a trusted source.

